# Medicine as a Moral Agent

**Published:** 1896-10

**Authors:** 


### Medicine as a Moral Agent.

Whereas formerly the sulky, stupid, or ill-tempered boy was commonly relieved of such distemper by the master's rod, it is now believed by a certain school of psychologists that with the judicious use of internal remedies his case is better reached than by this time-honored method of counter-irritation. A clearer insight would discover the salutary effects of a dose of castor-oil in many instances. The writer once knew the wife of a physician who habitually administered purgative doses of calomel to her two little boys with the sole intention of improving their dispositions. No less an authority than Dr. Lauder Brunton has directed the attention of the profession to the fact that many quick-tempered persons are really victims of masked forms of gout or rheumatism, and may be relieved by appropriate remedies which he has facetiously called *temper powders.—Medical News.*

